# Prospective randomized controlled trial study of Luofengning granule in the treatment of unstable angina

**DOI:** 10.1097/MD.0000000000020025

**Published:** 2020-05-15

**Authors:** Kai Wang, Jun-Jun Cai, Yang Wu, Yu Wang, Lei-Lei Liu, Lei Shi, Xian Wang

**Affiliations:** a Dongzhimen Hospital, Beijing University of Chinese Medicine, Beijing; b Department of Hepatology, Tianjin Third Central Hospital, Tianjin; c School of Traditional Chinese Medicine; d School of Life Science; e Institute for Cardiovascular Disease, Beijing University of Chinese Medicine, Beijing 100029, People's Republic of China.

**Keywords:** Chinese herbal medicine, Luofengning granule, randomized controlled trial, unstable angina

## Abstract

**Introduction:**

Although the current western treatment plans for unstable angina (UA) has been optimized in past decades, UA still is a common phenotype of acute coronary syndrome and significantly influence the quality of life and endanger lives. In China, the clinical application of Chinese herb medicine is considered as an effective approach to treating UA and widely recognized by patients. In clinical practices, we found Luofengning granule (LFN-G) could improve clinical manifestations of patients with UA, but there is lack of rigorous proof of evidence-based medicine. This trial aims to further evaluate the efficacy of LFN-G in the treatment of UA.

**Methods:**

A prospective, open-label, randomized, placebo-controlled clinical will be performed. A total of 60 patients diagnosed with UA will be randomly allocated to either the treatment group or the control group with a 1:1 ratio. The participants in the treatment group will receive LFN-G treatment and the participants in the control group will receive placebo. Meanwhile, both groups continue to undergo standard western medicine treatments. The duration of interventions is 4 weeks. The primary endpoint is the incidence of major cardiac adverse events, defined as a composite of recurrent angina, acute myocardial infarction (AMI), severe arrhythmia, heart failure, and cardiac death. Secondary outcomes include Seattle angina scale score, Chinese medicine syndromes and electrocardiograph (at weeks 0, 1, 2, 4), myocardial nuclides perfusion, measurement of wall motion score index and left ventricular ejection fraction, serum inflammation factors such as C-reactive protein, high sensitive-C-reactive protein, interleukin-6, matrix metalloproteinase-9, and so on (at weeks 0, 4). In addition, some biochemical indexes of blood and hematological indexes will be used to assess the safety of treatments. Any adverse effects of the treatment will be recorded.

**Discussion:**

The results of this trial will provide compelling evidence of the efficacy and safety of LFN-G for treatment of UA and preliminarily reveal the potential mechanism of how LFN-G acts. Finally, it will widen treatment options for patients with UA.

## 1 Introduction

Unstable angina (UA) is a phenotype of acute coronary syndrome (ACS), defined as myocardial ischemia at rest or minimal exertion in the absence of cardiomyocyte necrosis. Recent study indicated that the overall prevalence for angina is 3.4% in US adults ≥20 years of age.^[1]^ Approximately 10% of patients with acute chest pain present to the emergency department will be diagnosed with UA.^[2]^ Compared with the other 2 phenotypes of ACS, ST-elevation myocardial infarction (STEMI) and non-ST-elevation myocardial infarction (NSTEMI), UA exhibits a lower risk of in-hospital death.^[2,3]^ However, The Global Registry of Acute Coronary Events (GRACE) study showed there was no significant difference of 5-year total mortality among patients with 3 types of ACS (STEMI vs NSTEMI vs UA was 19% vs 22% vs 18%).^[4]^

Based on the pathogenesis of 3 types of ACS, the treatment of ACS exists differences. With vascular total occlusion caused by plaque rupture, coronary revascularization is the core of treatment of STEMI. In recent year, UA and NSTEMI were considered to present a similar clinical and electrocardiograph (ECG) features (ST-segment depressions and T-wave inversion). Clinical guidelines recommended rename the both referred to as the NSTE-ACS together and distinguishment based on high-sensitivity cardiac troponin measurements.^[2,5,6]^ In absence of coronary artery total occlusion, the treatment plans of NSTE-ACS based on the comprehensive evaluation based on clinical and ECG manifestations and myocardial biomarkers, including anti-myocardial ischemia therapy, antiplatelet or anticoagulation therapy, statin therapy, and revascularization therapy, and so on.

Although current treatment options of UA have been demonstrated for definite improvement of long-term prognosis, there still are some deficiencies. GRACE study showed post-ACS 5-year deaths most occurred after initial hospital discharge, post-discharge deaths occupied up to 97% of 5-year total deaths in UA and this figure was much higher than in STEMI (68%) and NSTEMI (86%).^[4]^ The cardiovascular prevention treatments after acute coronary syndrome (CPACS) study found only 31% of UA patients received revascularization therapy in China, which was lower than STEMI patients.^[7]^ Besides, some patients with UA manifest refractory angina, current treatments have shown some promise in angina reduction but not yet achieved the goal such as objective data like myocardial blood flow, survival, and re-hospitalization.^[8]^ These results may reflect insufficient attention were pay to the treatment of UA and current treatments for UA needs further optimization. Thus, complementary treatments for UA are urgently needed to further improve the prognosis and quality of life of patients with UA.

In addition to western medicine treatment, many Chinese patients with UA would like to seek help of Chinese herb medicine (CHM). CHM has been demonstrated to have a good clinical effect. Some meta-analysis has demonstrated the efficacy and safety of CHM.^[9,10,11]^ Luofengning granule (LFN-G) is a Chinese herbal formula based on the the theory of “endogenous wind due to collateral deficiency,” includes 6 herbs: Lumbricus (Di long), Radix Clematidis (Wei lingxian), Radix Cynanchi Paniculati (Xu changqing), Panax notoginseng (San qi), Astragalus (Huang qi), and Borneol (Bing pian). Previous observational study demonstrated that LFN-G has definite effectiveness in the treatment of UA^[12]^ associated with its’ multiple pharmacologic effects include anti-inflammatory,^[13]^ immune regulation,^[14]^ improving endothelial function,^[15]^ and coagulation regulation.^[16]^ However, there is no rigorous randomized controlled trial (RCT) that verify above conclusions. Thus, we design this prospective, open-label, randomized, placebo-controlled clinical to further evaluate the efficacy and safety of LFN-G for treating UA. This trial will provide a more compelling evidence of LFN-G and expand therapeutic options for UA.

## 2 Materials and methods

### 2.1 Ethical aspects

The study is conducted in accordance with the Declaration of Helsinki (Edinburgh 2000 version). The final protocol (version: November 31, 2019) of this trial has been approved by the Research Ethical Committee of Dongzhimen Hospital affiliated to Beijing University of Chinese Medicine (the ethical application has been approved). In addition, this trial has been registered in the Chinese Clinical Trial Registry (No. ChiCTR2000030533, registered March 4, 2020). Before the enrollment, all participants should sign written informed consents. If there will be any significant modification of the protocol, it should be reviewed by the research ethical committee and updated on the registry web (http://www.chictr.org.cn) timely.

### 2.2 Study design

**Figure 1:**
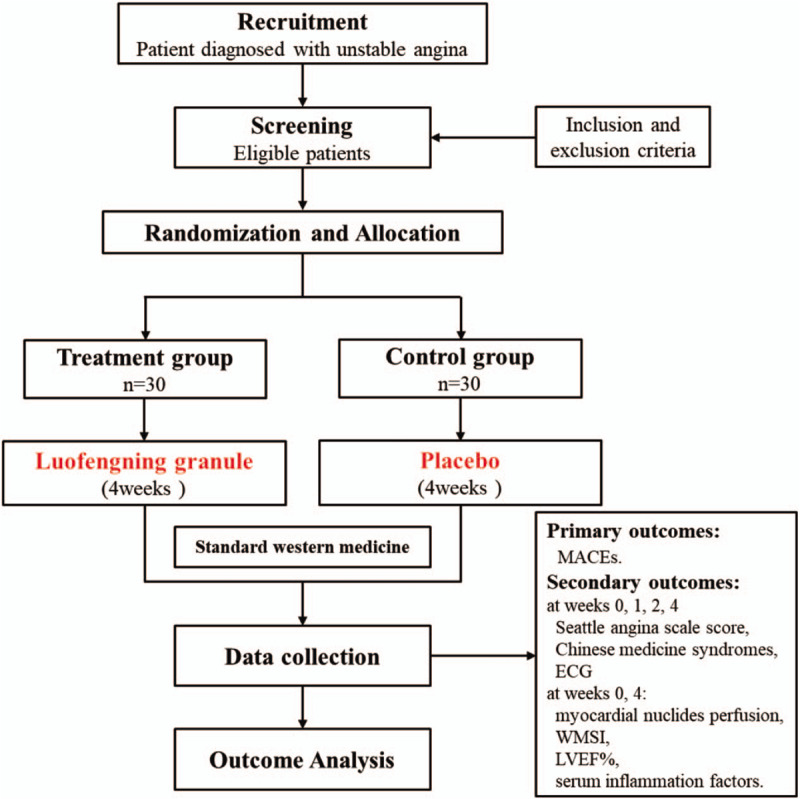
Flow chart of the study design. ECG = electrocardiograph, LVEF% = left ventricular ejection fraction, MACEs = major adverse cardiac events, WMSI = wall motion score index.

This present study is a prospective, open-label, randomized, placebo-controlled clinical trial. A total of 60 patients diagnosed with UA are eligible for enrolment and they will be randomly allocated to the treatment group or the control group in a 1:1 ratio. The study flowchart is showed in Figure 1.

### 2.3 Recruitment

Two trained investigators will perform this procedure. A total 60 patients will be recruited from cardiac out-patient and ward of the Dongzhimen hospital affiliated to Beijing University of Chinese Medicine, from June 2020 until December 2022. Before the enrollment, all patients will receive a comprehensive view of informed consent, including objective, therapeutic interventions, scheduling, trial benefits, and possible risks of this trial. Only after signing a written informed consent, patients could be enrolled in this trial.

### 2.4 Randomization and masking

A designated investigator of our team will use an interactive Web response system to randomly allocate participants to receive either LFN-G or placebo after enrollment in a 1:1 ratio. Randomization will be stratified by age, sex, morbid status, and comorbidities. Due to this trial is an open-label trial, so neither participants nor investigators is not masked to the treatment option.

### 2.5 Diagnostic criteria

Western medicine diagnostic criteria for UA were determined by referring to related guidelines for the management of NSTE-ACS.^[2,5,6]^ Traditional Chinese medicine (TCM) syndrome diagnostic criteria evaluation of UA is based on Standards for TCM industry of the People's Republic of China(ZY/T001.1–94).^[17]^ In the process of diagnosis and syndrome differentiation, 2 cardiac physicians of TCM will make the diagnosis respectively. If there exists diagnostic controversy, a consensus would be reached through further discussing or third-party advice.

### 2.6 Eligibility criteria

Inclusion criteria:

- Diagnosed with UA by a cardiac physician.
- 18 to 80 years old.
- No gender restriction.
- Voluntary to participate and providing written informed consents, as required by Good Clinical Practice (GCP).

Exclusion criteria:

- Angina pectoris caused by noncoronary stenosis such as abnormal thyroid function, cardiomyopathy, high adrenergic state, mental stress, left ventricular load increased (hypertension, aortic stenosis), tachycardia, violent activities, and so on.
- Patients with stroke or undergone PCI or coronary artery bypass grafting within 1 month before enrollment.
- Poorly controlled hypertension (systolic pressure ≥160 mm Hg or diastolic blood pressure ≥100 mm Hg).
- Patients with coexistent severe heart failure (New York heart association (NYHA), Class IV) and severe arrhythmia (atrial fibrillation, atrial flutter, paroxysmal ventricular tachycardia, sinus bradycardia [heart rate <55], complete left bundle branch block).
- Combined liver dysfunction (defined as Alanine transaminase (ALT) and/or Aspartate aminotransferase (AST) 3 times higher than the upper limit of normal) and kidney dysfunction (defined as creatinine clearance rate less than 30 mL/min/1.73 m^2^).
- The terminal phase cachexia of malignant tumors.
- High bleeding risk, such as hemorrhagic constitution, history of hemorrhage of vital organs (brain or upper gastrointestinal tract) within 6 months, low platelet counts, abnormal coagulation function, recent active hemorrhage, and so on.
- Underwent surgery within 1 month.
- Women who are under breastfeeding, pregnancy or preparing for pregnancy.
- Poor compliance.
- Known or suspected to be allergic to experimental drugs or allergic constitution.
- Patients who participate in any other clinical study or take any of its investigational drugs within the last 3 months.

### 2.7 Termination and withdrawal criteria

All participants have right to withdraw from the trial depends on their own will. The reason will be recorded in the case report forms (CRFs) and the patient will continue standard western medicine treatment after withdrawing. The criteria of termination and withdrawal including:

- The participant who has serious adverse events (AEs) may caused by experimental drug is considered to be inappropriate to continue the trial based on the cardiologist's judgment.
- The participant's condition becomes deteriorated or develops other diseases that may affect the study.
- Important deviations occur in the process of the clinical trial, such as poor compliance.
- The participant is not willing to continue the clinical trial.

### 2.8 Test drugs

Drugs for clinical trials are LFN-G and placebo (LFN-G mimics), provided by the Guangdong E-Fong Pharmaceutical Technology Development Co, Ltd (Guangdong, China). Formula ingredients of LFN-G includes: Lumbricus (Di long) 10 g, Radix Clematidis (Wei lingxian) 15 g, Radix Cynanchi Paniculati (Xu changqing) 12 g, Panax notoginseng (San qi) 3 g, Astragalus (Huang qi) 10 g, and Borneol (Bing pian) 0.1 g. All ingredients are herbal granules. These granules are mixed and packaged into 2 single-dose sachets, each weighing 25.05 g. The placebo consists of medical starch dextrin without active ingredients. Through adding a variety of food colorings and flavouring agents, the placebo will present a similar weight, appearance and taste as close as possible to the real LFN-G.

## 3 Treatment

### 3.1 Treatment plan

All participants will receive a standard western medicine treatment in accordance with guidelines^[2,5,6]^ judged by a senior cardiology physician, including but not limited to anti-myocardial ischemia therapy, antiplatelet or anticoagulation therapy, statin therapy. Meanwhile, all participants will accept lifestyle adjustments.

Experimental group: Participants will receive LFN-Gs (divided into 2 equally- weight in separate packages, each 25.05 g), dissolving each package in 100 mL warm boiled water after breakfast and supper for 4 weeks.

Control group: Patients will be given placebo granules (divided into 2 equally- weight in separate packages, each 25.05 g). The administration is in accordance with the experimental group.

### 3.2 Collection of serum

The subject blood samples will be collected by a designated nurse before and after intervention and designated investigator will centrifuge blood samples in the laboratory. After centrifugation, the supernatant was transferred in frozen pipe and stored at −80°C for preservation in the biological sample bank of the Dongzhimen hospital. All samples will be destructed when the trial end.

### 3.3 Outcome measures

Primary outcome is the incidence of major cardiac adverse events, defined as a composite of recurrent angina, acute myocardial infarction, severe arrhythmia, heart failure, and cardiac death. This outcome will be compared with the baseline and the 4th week.

Secondary outcomes including:

(1) Seattle angina scale score: This score includes 4 aspects (physical activity limitation, angina frequency, treatment satisfaction, and disease cognition) and it can provide information of participants’ subjective assessment of therapy. This outcome will be compared the baseline with the 1st, 2nd, 3rd, and 4th week.
(2) Chinese medicine syndromes: This outcome will be compared the baseline with the 1st, 2nd, 3rd, and 4th week.
(3) ECG: The change of St-segment and T wave of baseline and the 1st, 2nd, 3rd, and 4th week.
(4) Myocardial nuclides perfusion: It will be used to evaluate myocardial perfusion under endocardium and epicardium of the baseline and the 4th week.
(5) Echocardiography: Measurement of wall motion score index and left ventricular ejection fraction of the baseline and the 4th week.
(6) Vitro measurement: Measurement of serum inflammation factors such as C-reactive protein, high sensitive-C-reactive protein, interleukin-6, matrix metalloproteinase-9, and so on.

### 3.4 Safety assessment

The dosage of experimental herbs used in this trial is all within the recommended range based on the People's Republic of China Pharmacopeia (2015 edition). At the process of trial, we also utilize laboratory tests to evaluate liver and kidney function to ensure the safety of participants from the time of enrollment through the follow-up period.

### 3.5 AEs

Any AEs will be requested to record in CRFs regardless of their relationship to the intervention. If serious AEs occur, the intervention should be stopped immediately and a detailed description of the time, severity, relationship with the drug, and the treatment measurement will be recorded. Serious AEs will be reported to the Steering Committee and Ethics Committee within 24 hours. In addition, a follow-up will be conducted until the AEs disappeared.

### 3.6 Data management and quality control

All information related with this trial will be recorded in CRFs by a trained and qualified investigator. No correction of completed CRFs is allowed once completed. The record of CRFs will be reviewed by the clinical inspector. To ensure the accuracy of the data, 2 investigators will input and proofread the data respectively. In addition, all papery documents and electronic versions of the CRF will be preserved in the secure research archives for 5 years after trial completing at the Dongzhimen Hospital Affiliated to Beijing University of Chinese Medicine and only can be viewed by the research team.

### 3.7 Statistical analysis

The data will be analyzed using the Statistical Package for the Social Sciences version 22.0 (SPSS 22.0, Chicago, IL). The qualitative data is analyzed by descriptive statistics, expressed by frequency and percentage; the measurement data is expressed as mean ± standard deviation and tested by *t* test or *q* test. Chi-square test is used for counting data. Spearman correlation analysis is used for single factors and logistic regression analysis is used for multiple factors. The composition ratio data is tested by *x*^2^ test. Unidirectional ordered data were tested by rank sum test. All statistical tests will be bilateral tests and *P*-values < .05 is considered to indicate statistical significance.

## 4 Discussion

UA is a common manifestation of coronary artery disease that seriously affects the quality of life and causes deaths and potential years of life lost. Current treatments for UA are still not satisfy all clinical conditions. There is a wide acceptance of CHM for treating coronary artery disease in China and it has obvious curative effect indeed. In a long-term clinical work of our team, we have found the efficacy and safety of LFN-G in treatment of UA. But we need a compelling RCT evidence to further demonstrate the benefits and risks of adding LFN-G to the standard western medicine treatment for patients with UA.

In order to evaluate the efficacy and safety of LFN-G, we design this prospective, open-label, randomized, placebo-controlled trial. In this trial, we focus on the incidence of major cardiac adverse events, angina score, quality of life, and utilize some objective physical and chemical indicators to ensure the reliability and generality of the results.

There exist some limitations to this study. First, this is a single-center, small scale RCT. Second, the follow-up duration is relatively short. Third, this trial will be performed in Beijing, China, there may be regional and ethnic deviations. Thus, if we finally get a promising result from this current, we consider conducting a multicenter, randomized, large scale, and long follow-up trial in order to provide more significant information to guide clinical treatment.

## Author contributions

**Conceptualization:** Xian Wang.

**Investigation:** Kai Wang, Yang Wu, Yu Wang.

**Project administration:** Xian Wang.

**Methodology:** Lei-Lei Liu, Lei Shi.

**Project administration:** Xian Wang.

**Trial design:** Kai Wang, Xian Wang.

**Writing – original draft:** Kai Wang, Jun-Jun Cai.

**Writing – review and editing:** Lei-Lei Liu, Lei Shi, Xian Wang.
